# Evaluating the Effectiveness of an Enhanced Early Childhood Development Program Integrated Into Primary Health Care in China: Protocol for a Cluster Randomized Controlled Trial

**DOI:** 10.2196/89106

**Published:** 2026-05-27

**Authors:** Yunting Zhang, Yuanyuan Dong, Haiwa Wang, Huan Zhou, Guohong Li, Zhongxun Dong, Jiali Jiao, Yuju Wu, Shaofang Qi, Zhangsheng Yu, Xiaolin Wei, Fan Jiang

**Affiliations:** 1 Department of Developmental and Behavioral Pediatrics, Child Health Advocacy Institute and Shanghai Key Laboratory of Child Brain and Development, National Children's Medical Center, Shanghai Children's Medical Center, Shanghai Jiao Tong University School of Medicine, 1678 Dongfang Road, Shanghai, 200025, China, 86 21 3808 7950; 2 West China School of Public Health and West China Fourth Hospital, Sichuan University, Sichuan, China; 3 School of Public Health, Shanghai Jiao Tong University School of Medicine, Shanghai, China; 4 Institute of Clinical Medicine, Shanghai Jiao Tong University School of Medicine, Shanghai, China; 5 Center for Biomedical Data Science, Institute of Translational Medicine, Shanghai Jiao Tong University, Shanghai, China; 6 Dalla Lana School of Public Health, University of Toronto, Toronto, ON, Canada

**Keywords:** early childhood development, primary health care, cluster randomized controlled trial, study protocol, effectiveness

## Abstract

**Background:**

Early childhood development (ECD) programs refer to policies and programs aimed at protecting young children’s rights to achieve their full potential. Parenting interventions are effective at improving children’s cognitive development and overall well-being. However, there is limited evidence on how to effectively implement and integrate such programs into routine service delivery at scale. The Government of China launched the ECD program in 2013 and the ECD scale-up program in 2023.

**Objective:**

Our study aims to design, implement, and evaluate an enhanced ECD program embedded in the primary health care system in order to provide ECD policy recommendations for future national scale-up.

**Methods:**

This study involves a multicenter cluster randomized controlled superiority trial in 3 counties with different levels of socioeconomic development in China. Fifty-eight clusters will be randomly assigned in a 1:1 ratio to intervention and control groups, stratified by county. In each cluster, 18 families with children aged 6-23 months will be recruited. Our intervention aims to implement an enhanced ECD program in primary care facilities. Intervention group participants will receive usual care alongside enhanced ECD services, including improved facilities, capacity building, and improved ECD services (age-adapted clinical consultations on nutrition and stimulation, age-specific parenting group sessions, home visits, and referrals for children in need). Moreover, an electronic data platform will be used. Control group participants will receive usual care. Our primary outcome is children’s overall development, as measured by the Global Scale of Early Development combined form at 12 months of follow-up. Evaluation data will be collected in both arms. Additionally, a process evaluation and an incremental cost-effectiveness analysis will be conducted. Primary analysis will follow the intention-to-treat principle, and it will use generalized estimating equations to assess the population average intervention effect, with adjustment for corresponding baseline outcomes, selected individual variables, county, cluster variables, and variables differing at baseline. Qualitative data will be collected through semistructured interviews and focus groups, guided by the Capability, Opportunity, Motivation, and Behavior framework to explore implementation-related factors. Data will be transcribed for analysis using NVivo 14 (Lumivero). Ethical approval has been obtained from the Institutional Review Board of Shanghai Children’s Medical Center Affiliated to Shanghai Jiao Tong University School of Medicine.

**Results:**

This study was funded in 2023 and registered in April 2024. The recruitment of 1044 participants was completed on September 14, 2024. Data collection was completed on January 31, 2026.

**Conclusions:**

We aim to design an enhanced ECD service delivery model that can be embedded into the routine primary health care system. The study findings will guide government policies and reforms for strengthening the health system to scale up ECD services. Furthermore, this project may provide policy evidence to support countries in establishing or expanding their ECD services.

## Introduction

### Background

Early childhood development (ECD) programs include policies and programs aimed at protecting young children’s rights to develop their full cognitive, emotional, social, and physical potential—crucial to the well-being of children, communities, and nations. It was estimated that over 43% of children aged younger than 5 years in low- and middle-income countries were at risk of poor development in 2010 [1]. Moreover, existing evidence indicates significant inequalities in early experiences and outcomes. Disparities in development during the early years tend to persist throughout the life course and may even be carried into future generations [2]. The Nurturing Care Framework serves as a foundational framework, highlighting that opportunities for stimulation, responsive parent-child interactions, and positive parenting are crucial for children’s early development [3]. Several systematic reviews consistently demonstrated a moderate positive impact of parenting interventions on improving children’s cognitive development [4-6]. These interventions are delivered through home visits, group activities, or clinic consultations [4,7], with modalities adapted to local contexts, and have consistently been proven to be effective in improving parenting behaviors and child development [5,8].

All evidence points to the need to scale up ECD interventions to a larger population [9-11]. Some countries have already pioneered the expansion of ECD projects nationwide. Brazil was the first country to adopt a nationwide ECD program, the Criança Feliz Program (happy child), in 2016, providing home visits by primary health care workers to enhance caregivers’ parenting skills [12]. In Peru, the Cuna Mas program was started in 2012 and expanded to provide home visits to over 67,000 low-income families in rural areas, making it one of the most extensive programs of its kind [13]. Despite their positive impact on children’s development, both programs faced implementation challenges [12,13], such as low attendance rates and high facilitator turnover, regardless of whether the personnel were community health workers or recruited home visitors. More importantly, as pointed out by Brentani et al [12], the Brazil program is organized in a highly decentralized way, with the federal government providing funding to participating municipalities, and many questions regarding implementation remain unanswered at the municipal level. Recent reviews and studies on parenting interventions also pointed out that reporting on implementation characteristics is poor and inconsistent, limiting efforts to replicate, adapt, and scale up interventions [4,10,14,15].

Embedding parenting interventions into existing pediatric primary care, such as well-baby checkups, may help address these challenges. Recent meta-analyses have highlighted the unique roles played by pediatricians in ECD interventions, given their specialized knowledge and ongoing relationships with families [16,17]. Several pediatric primary care–based parenting interventions, such as Healthy Steps, Play Read VIP, and Reach Out and Read, have demonstrated effectiveness and scalability [16,17]. However, most of these programs rely on child development specialists or health care professionals, whose availability is limited in low-resource settings. This raises important implementation challenges for adapting and replicating such interventions within primary health care systems across regions with varying developmental stages.

China, as an upper-middle–income country, is facing significant challenges in ECD due to substantial economic disparities across the country. In the past 70 years, the child health care system played a unique role in reducing child mortality and malnutrition rates across the nation [18]. As part of the system, child health providers in community or township primary care facilities are responsible for conducting regular health checkups for children aged 0‐3 years. They work with health workers/village doctors in subordinate community health service stations or village clinics. The primary care team is under the supervision of hierarchical maternal and child health (MCH) centers from the county level to the national level [19]. These MCH institutes have 3 major functions: management and regulation, clinical treatment, and public health service provision. The higher-level MCH institutes supervise the lower-level MCH institutes. MCH institutes at the district or county level coordinate all MCH services, including general hospitals and specialist hospitals [20].

Since the MCH system is the most integrated system for managing the health of children aged 0-3 years in China, implementing and scaling ECD services through this system is the most feasible approach. Specifically, ECD services could be provided along with current regular health checkups to achieve population-level coverage. The Government of China started the ECD program in 2013. In 2023, the National Health Commission partnered with the National Working Committee on Children and Women under the State Council and National Rural Revitalization Administration to launch an ECD scale-up program in 192 counties across 30 provinces [21]. Our aim is to design and implement an ECD program that is effective and fits into the diverse developmental levels in China.

Compared with existing primary care–based ECD programs that typically rely on child development specialists or intensive physician involvement [16,17], the Chinese MCH system achieves universal coverage through a frontline workforce of health workers and village doctors [22]. This creates a distinct implementation challenge: ensuring adequate intervention dosage within a system characterized by limited provider time but standardized [23], frequent health checkups (6 times in year 1 and biannually thereafter). With each visit lasting under 15 minutes, there is insufficient time for comprehensive parenting skills training. To address this, we propose leveraging the MCH system’s structured schedule and population coverage by integrating brief ECD counseling into routine health checkups and supplementing the approach with more frequent parenting group sessions outside the clinic for hands-on coaching. Critically, rather than relying on external research teams for training and supervision, we utilize the MCH system’s inherent hierarchical structure to build sustainable implementation capacity [24]. Lastly, to evaluate the generalizability of this model across diverse economic contexts, this study uses a multicenter design spanning regions at different developmental stages.

### Objectives

In this study, we will design, implement, and evaluate an enhanced ECD program embedded in primary care in China, using a hybrid effectiveness/implementation trial, a process evaluation, and an economic cost-effectiveness analysis. We aim to (1) evaluate the effectiveness of the enhanced ECD program, (2) identify implementation problems and inform implementation interventions, and (3) assess the cost of this enhanced ECD program.

## Methods

### Study Design

This protocol is reported according to the SPIRIT (Standard Protocol Items: Recommendations for Interventional Trials) guidelines [25]. The SPIRIT checklist is provided in Checklist 1. This study involves a multisite cluster randomized controlled superiority trial with 1 intervention and 1 control arm, using 1:1 allocation stratified within each county. The study design is informed by the Medical Research Council Framework [26] on complex interventions and implementation science frameworks, with an embedded theory-based process evaluation to examine operational questions regarding feasibility and acceptability. We have used the Theoretical Domains Framework [27] to explain implementation problems and inform implementation interventions [26]. The intervention will last for 1 year. Recruitment and baseline surveys were conducted from July to September 2024, with data collection for endpoint evaluation to be concluded in January 2026. Process evaluations are scheduled at 3 and 9 months after baseline. An incremental cost-effectiveness analysis will also be conducted.

### Setting

The study will be conducted in 3 counties across China: Yiwu (Zhejiang Province), Xinmi (Henan Province), and Zhijin (Guizhou Province). The 3 counties are situated in the eastern, central, and western regions of China, reflecting a gradient of economic development. The gross domestic product values of residents per capita in 2023 for Yiwu, Xinmi, and Zhijin were US $14,862, $12,348, and $4509, respectively [28-30]. In addition to differences in economic development, in 2023, Yiwu, Xinmi, and Zhijin had mortality rates among children aged younger than 5 years of 2.4%, 2.67%, and 3.07%, respectively.

### Eligibility and Recruitment

Families of children aged younger than 3 years regularly receive health checkup services from community or township primary care facilities. Children will be considered for inclusion in this study if they (1) are aged 6‐23 months; (2) are born at full term (≥37 weeks of gestation; singleton pregnancy) without apparent diseases (healthy); (3) have a primary caregiver aged ≤65 years (as older caregivers may find it difficult to take children for parenting group activities every other week and may face challenges in using eHealth) with no plan to move away from the local community in the next 12 months; (4) have a primary caregiver who indicates willingness to participate in the program and follow-up surveys at specified times; and (5) are residing within a 2-km radius of the community or township primary care facilities (extension to 4 km or further if insufficient candidates are available).

Children will be excluded if they (1) have a birth weight below 2500 grams; (2) have severe birth defects, genetic diseases, or inborn errors of metabolism; (3) have severe infectious diseases, neonatal hyperbilirubinemia, neonatal convulsions, persistent hypoglycemia, and other such issues in the neonatal period; (4) have severe birth injuries requiring hospitalization; (5) are diagnosed with global developmental delay by an authoritative agency; (6) have any other condition that affects growth or development; and (7) have a primary caregiver diagnosed with mental or neurological diseases (eg, depression and schizophrenia).

In China, parents are generally reluctant to take very young infants out of the home, which may reduce participation in group activities. The lower age limit of 6 months reflects this consideration, and the upper age limit of 23 months ensures that all children remain younger than 3 years at the 1-year endpoint. Only 1 child per family can be recruited in the study. Once the list of eligible children in each community or township is acquired, a statistician will reorder the list using a randomization algorithm to ensure that each child has an equal opportunity to participate. Child health providers will recruit children sequentially from this list, confirming the ability and willingness of caregivers to participate in parenting group activities every other week. All participants in the same cluster will be assigned to the same study arm. Services provided in this study will be available to all children aged 0‐3 years in the intervention arm, but data will be collected only from children formally enrolled in the trial.

### Randomization and Blinding

A total of 58 clusters (ie, communities or townships) will be randomly assigned in a 1:1 ratio to the intervention group or control group, stratified by county. A block randomization method with a random block size of 4 or 6 will be used. The allocation sequence will be generated by a statistician and implemented using sequentially numbered lists. Child health providers in each cluster will enroll participants based on the random sequence. The participants in each cluster will be randomly selected according to the inclusion and exclusion criteria. A total of 18 children will be enrolled in each cluster, with 6 children in each age stratification group (6‐11, 12-17, and 18‐23 months). Participants in the intervention clusters will receive enhanced ECD services and regular health checkups. Participants in the control group will receive regular health checkups.

Participants, researchers, and implementation stakeholders will not be blinded to the intervention. However, evaluators evaluating study outcomes will remain blinded to group allocation.

### Procedures

#### Control Arm: Usual Care

Children aged younger than 3 years receive regular health checkups at community or township primary care facilities. In accordance with national public health service standards, health checkups are scheduled at the following ages: 7 days, 1 month, 3 months, 6 months, 8 months, 12 months, 18 months, 24 months, 30 months, and 36 months. At each visit, a child health provider conducts a growth evaluation, physical examination, and brief developmental screening. They also provide anticipatory guidance to caregivers. If a disorder or disease is identified or suspected, the child is referred to a higher-level MCH institute for further diagnosis and treatment. Child health providers maintain records for all children aged younger than 3 years in their management area, notify parents of upcoming visits, and follow up with those who miss appointments. As of 2023, over 90% of children received health checkups as scheduled in all the 3 counties.

Participants in the control group will receive usual care, which includes regular health checkups without enhanced ECD services. Child health providers will offer consultations in an unstructured manner without using a nurturing care flipboard or the eHealth program. In Zhijin and Xinmi County, no parenting group facilitators exist in community or township primary care facilities, and there are no parenting group activities or home visits to enhance parenting skills. Yiwu County, the most developed county, has implemented self-administered parenting group activities in primary care facilities, where parenting group sessions are provided quarterly, which is significantly less frequent than in the intervention arm. There are no connections between the parenting group facilitators and child health providers in primary care facilities. A comparison of the components in the intervention and control groups is presented in Table 1.

**Table 1.** Comparison of components between the intervention and control groups.

Component	Intervention group (primary care–based enhanced ECD^a^ program)	Control group (usual care)
Facilities and personnel	Presence of an ECD center in community or township primary care facilities. The team includes child health providers, parenting group facilitators, village health workers, and general practitioners.	In community or township primary care facilities, child health providers and village health workers collaborate to deliver preventive child health care, operating independently from general practitioners. In Yiwu, parenting group facilitators work independently alongside health providers. However, in Xinmi and Zhijin, there are no parenting group facilitators.
Services	Clinical consultation: Utilize a standardized checklist to identify risks related to nutrition and stimulation, and provide guidance to parents based on the Nurturing Care Framework. Parenting group activity: Conduct age-specific parenting sessions every other week, offering health education and demonstrations on parent-child interactions, with a focus on responsive care. Home visit: Enhance parenting skills for families with low attendance at parenting group sessions, which is provided in collaboration with parenting group facilitators and village health workers. Provide health examinations and consultations to families with low attendance at regular health checkups. Referral: Involve general practitioners in the diagnosis and treatment of children with medical conditions.	Clinical consultation: Conduct health checkups paired with spontaneous consultations. Parenting group activity: In Yiwu, parenting sessions are offered to children in a simplified approach, which consists of only 4 sessions per year, with parental participation being voluntary. In the other 2 counties, there is no availability at community or township primary care facilities. Home visit: Not provided.
eHealth	Parents can receive individualized parenting guidance and schedule appointments for parenting group activities via their mobile devices.	No eHealth programs.
Training	Provide joint training for both child health providers and parenting group facilitators in ECD concepts and skills. Enhance consultation skills using the “Care for Child Development” package.	Training for child health providers and parenting group facilitators is conducted separately, and there is insufficient emphasis.
Supervision	Child health providers are responsible for monitoring all enhanced ECD services and providing technical support to parenting group facilitators and village health workers.	Child health providers are responsible for maintaining records of regular health checkups.

a ECD: early child development.

#### Intervention Arm: Enhanced ECD Program

##### Facilities and Personnel

We will provide an enhanced ECD program integrated into primary care settings. We will establish an ECD center in each community or township primary care facility in the intervention arm. At each ECD center, child health providers will work with 4 additional parenting group facilitators, who will be part-time workers selected from among family planning specialists, village health workers, preschool teachers, and peer mothers in the community. As of March 31, 2026, 1.8% (2/113) of parenting group facilitators had completed junior high school, 15.0% (17/113) had graduated from high school or secondary vocational school, and 83.2% (94/113) held a college degree or higher. A general practitioner at the township/community level will participate in the center. At the village level, the village health workers will collaborate with parenting group facilitators to provide home visits. The responsibilities of each role are listed in Table 2.

**Table 2.** Roles of health personnel providing the ECD^a^ program.

Personnel	Roles
Child health providers	Being the key person responsible for ECD-based health management Providing ECD counseling services starting from the first health checkup, in addition to regular health checkups Working with parenting group facilitators to arrange parenting group activities Providing technical support to parenting group facilitators and home visitors Providing training to home visitors Participating in quality control of the parenting group activity and home visit
Parenting group facilitators	Carrying out parenting group activities Referring children who show any medical or developmental issues to child health providers or general practitioners Carrying out home visits
Village health workers	Carrying out home visits Referring children who show any medical or developmental issues to child health providers or general practitioners
General practitioners	Providing clinical services to children with medical conditions

a ECD: early child development.

##### Services

For children undergoing regular health checkups, child health providers will use an age-adapted consulting checklist to identify risks related to nutrition and stimulation (details are provided in Multimedia Appendix 1).

A flipboard will guide the child health providers in consulting parents on all 5 domains of the Nurturing Care Framework: health, nutrition, responsive caregiving, safety and security, and early stimulation. If any risks are identified, child health providers will explain the relevant knowledge and skills to caregivers.

During health checkups, child health providers will introduce parents to the parenting group activity. Compared with consultations in clinics, this newly added service offers more frequent sessions and expanded opportunities for on-site demonstrations and practice. A total of 56 sessions have been designed for children aged 2‐36 months. Families will be scheduled for parenting group sessions every other week before the age of 24 months and every month thereafter. Sessions will take place in training rooms (area of more than 15 m²) located within the primary care facilities. Each session will be led by 2 parenting group facilitators and host 5‐10 children of a similar age and their caregivers. For children aged younger than 12 months, the age difference of the children in the group session was maintained within 2 months, and for children aged 12-36 months, the age difference was maintained within 3 months. Each session will deliver both health education and demonstrations on responsive caregiving, concluding with the sharing of parenting experiences among all participants.

As an operational unit, child health providers and parenting group facilitators will meet monthly to discuss approaches for continuous improvement. The group will also discuss concerns regarding children with potential developmental risks or medical conditions detected during group sessions. Child health providers will refer children with further clinical needs to general practitioners.

For families who are willing to participate in the program but face challenges in attending sessions at the center regularly, for example, due to illness, transportation issues, or caregiving constraints, child health providers will notify parenting group facilitators and village health workers to arrange joint home visits. These visits will serve as a complementary component of the intervention. They are intended to support families with low attendance, rather than being a core element of the program delivered to all participants.

##### eHealth for Parents

An electronic system has been designed to support all enhanced ECD services provided during health checkups and parenting group activities. For clinical consultations, a checklist to identify nutrition and stimulation risks will be embedded in the child health provider’s account. Responses to the questions will be recorded to generate tailored recommendations. Parents will be able to scan a QR code to acquire their individualized recommendations or receive them directly on their own eHealth program account so that they can review and share them with other caregivers after they return home.

Families will be able to use the eHealth program to make appointments and receive reminders for parenting group activities. They will also check in and check out of each session using their own account.

##### Training

Child health providers will receive training on clinical consultations and parenting group activities to ensure they fully understand parenting knowledge and the procedures. Parenting group facilitators will also receive training. All training sessions, adapted from the Care for Child Development package, will focus on supporting parents during consultations and group sessions. Child health providers will also train home visitors for home visiting services.

##### Supervision and Monitoring

Child health providers will supervise 1 session per week to maintain parenting group facilitators’ skills in providing supportive and encouraging guidance to parents. The eHealth program will record attendance at clinical consultations and parenting group activities. A reporting system will allow child health providers to track the services received by each parent and address any deviation from the schedule. Additionally, child health providers will be able to use the program to monitor parenting group facilitators’ workload.

**Figure 1.**
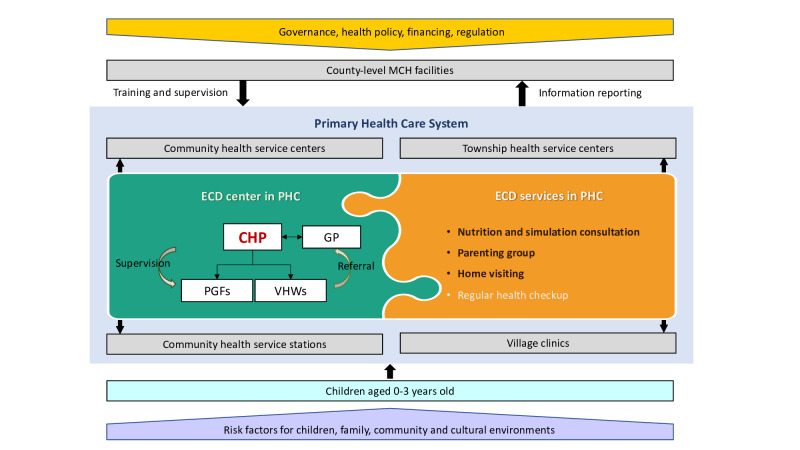
Primary care–based enhanced early childhood development (ECD) program. CHP: child health provider; GP: general practitioner; MCH: maternal and child health; PGF: parenting group facilitator; PHC: primary health care; VHW: village health worker.

Overall, the primary care–based ECD program will provide enhanced ECD services in primary health settings, including community/township primary care facilities and community health service stations/village-level clinics. The model will be integrated into existing MCH systems to address risks and promote ECD for children aged 0‐3 years (Figure 1). To our knowledge, no other ECD interventions will be implemented during our study.

### Outcomes

The primary outcome is children’s overall development measured using the Global Scale of Early Development (GSED) combined form. The GSED is an open-access package specifically designed to provide a standardized method for measuring child development up to 36 months at the population level globally [31]. The GSED package includes a long form administered directly to children, as well as short forms completed by caregivers. The combined form consists of all items drawn from both the long and short forms [32]. The full GSED package will be administered by certified assessors at baseline and the endpoint. All assessors will be required to achieve >90% score consistency with trainers before certification. Children’s overall development will be measured using a standard scale, the developmental score (D-score) [33], and the obtained score will then be transformed into an age-standardized score, the development-for-age *z* score (DAZ). The DAZ calculated from the combined form will serve as the primary outcome.

The secondary outcomes include children’s growth and nutritional status, as well as parents’ feeding and parenting practices associated with the enhancement of their knowledge, self-efficacy, and behavior. Furthermore, we aim to examine whether the intervention improves the family environment and functioning, as well as caregivers’ mental health and quality of life (Table 3). Therefore, the secondary outcomes are as follows: (1) children’s overall development measured by the DAZ (calculated from the short and long forms separately); (2) children’s developmental delay evaluated using the Ages and Stages Questionnaire third version (ASQ-3) and Parent-Reported Indicator of Developmental Evaluation for Chinese Children (PRIDE) [34]; (3) length-for-age *z* score, weight-for-age *z* score, weight-for-length *z* score, and stunting and wasting rates assessed through anthropometric measurements of the children; (4) anemia rate assessed through children’s hemoglobin levels (g/dL); and (5) other caregiver-reported measures, including feeding practices, parenting knowledge, self-efficacy, skills and behaviors, family environment, and functioning, as well as the mental health and quality of life of the primary caregiver (Table 3).

**Table 3.** Caregiver-reported outcomes.

Outcome of interest	Indicators and measures
Feeding practice	Percentage of children receiving qualified feeding based on diversity and frequency assessed using a checklist with 4‐9 binary indicators on breastfeeding, diet diversity, complementary feeding, and food supplements.
Parenting capability, motivation, opportunity, and behaviors	
Parenting capability of the primary caregiver	Scores for each domain measured via a self-administered questionnaire based on the COM-B^a^ model of behavioral science.
Parenting opportunity of the primary caregiver	Scores for each domain measured via a self-administered questionnaire based on the COM-B model of behavioral science.
Parenting motivation of the primary caregiver	Scores for each domain measured via a self-administered questionnaire based on the COM-B model of behavioral science.
Parenting behavior of the primary caregiver (integrated family parenting environment）	Scores assessed using the Family Care Indicators scale.
Family outcomes	
Family discipline behaviors	Percentage of children experiencing physical punishment within a month according to the Multiple Indicator Cluster Surveys.
Family function	Scores assessed using the Family APGAR^b^ Questionnaire.
Children’s screen exposure	Hours of screen exposure on weekdays assessed using a self-administered questionnaire.
Mental health and life quality of the caregiver	
Mental health of the caregiver	Scores assessed using the Center for Epidemiology Depression Scale.
Life quality of the caregiver	Scores assessed using the EQ-5D-5L.

a COM-B: Capability, Opportunity, Motivation, and Behavior.

b APGAR: Adaptation, Partnership, Growth, Affection, Resolve.

### Sample Size

Based on the desired effect size and cluster randomization, a total of 1044 children across 58 clusters will be enrolled.

There are a total of 65 clusters (communities/townships) across the 3 counties. Initially, we identified the number of children aged 6‐23 months in each cluster. We excluded 4 clusters with fewer than 30 children, as conducting parenting group activities would be impractical. Additionally, 3 clusters were excluded due to their involvement in other parenting training projects. This left 58 clusters for inclusion, with 29 clusters per arm, including 7 clusters from Yiwu, 8 from Xinmi, and 14 from Zhijin.

We calculated the required sample size using the formula for cluster randomized controlled trials with a fixed number of clusters, as described by Hemming et al [35]. As no published studies have reported effect sizes using the GSED, we drew on evidence from studies using other validated developmental assessment tools (eg, Bayley Scales of Infant and Toddler Development, Gesell Development Schedules, and ASQ-3). This approach is supported by evidence showing that the GSED D-score is highly correlated with the Bayley cognitive score (Pearson *r*=0.97) [36]. A recent meta-analysis on 65 parenting interventions globally estimated an effect size of 0.31 SD for improving cognitive development [4]. Another meta-analysis focusing on parenting training projects in the rural areas of China suggested an average effect size of 0.26 SD for improving children’s cognitive abilities [37]. Based on this, we anticipated a difference of 0.26 SD in children’s overall development. To inform the sample size calculation for this cluster randomized trial, we estimated intracluster correlation coefficients (ICCs) for our primary outcome (the Bayley-III cognitive composite score) at both the village and township levels. These estimates were derived from historical data of 4391 children aged 6‐36 months assessed in the rural areas of 3 Chinese provinces between 2015 and 2019 [38-40]. Using multilevel mixed-effects models in Stata 17.0 (StataCorp; controlling for the Bayley tester, province, and study fixed effects), we obtained the following ICCs for the change in scores from baseline to follow-up: 0.067 at the village level and 0.060 at the township level. As our trial was cluster randomized at the township level, an ICC of 0.06 was applied in the calculation. A sample size of 15 per cluster was estimated. Taking into consideration an attrition rate of 15%, a sample size of 18 children per cluster and a total sample size of 1044 were estimated to achieve 80% power with an α of .05.

### Pilot Stage

We assessed the feasibility of implementing the intervention through a pilot study in Yiwu, which served as the starting point for the project among the 3 counties. Specifically, we first tested the procedure for recruiting children and families and conducting baseline evaluations. Following this, we took 2 weeks to assess the feasibility and acceptability of (1) using the electronic platform to consult parents in regular health checkups; (2) arranging parenting group activities every other week; (3) utilizing the eHealth program for caregivers to receive enhanced ECD services; and (4) conducting supervision and monitoring at the community or township level. During the pilot, we strictly followed the recruitment and implementation protocols. One issue identified was the need for clearer guidance for health providers on supervising parenting group activities. To address this, we developed a detailed checklist for supervisors to assess the fidelity of the intervention (details are provided in Multimedia Appendix 2).

### Process Evaluation

We will use the RE-AIM (Reach, Effectiveness, Adoption, Implementation, and Maintenance) framework to measure implementation indicators, including reach, effectiveness, adoption, implementation, and maintenance [41,42] (Table 4), in order to assess the implementation process. The indicators will be collected through our program’s electronic monitoring system, questionnaire surveys, and qualitative interviews. With validated implementation measures, 3 key domains, including acceptability, feasibility, and sustainability of the intervention, will be evaluated from the perspectives of both participants and providers through questionnaire surveys and qualitative interviews [43,44].

**Table 4.** Description of dimensions and relevant indicators in process evaluation based on the RE-AIM^a^ framework.

Dimension	Indicators
Reach	Characteristics of the participating target children and caregivers (eg, age, sex, distance to the health center, education level, and family economic level) Characteristics of nonparticipants Attendance rates: percentage of target children receiving outpatient personalized counseling services and percentage of target children participating in parenting group activities
Effectiveness	Caregivers’ satisfaction with the implementation process Caregivers’ parenting knowledge and behaviors during the implementation process Changes in the knowledge and skills of child health providers and parenting group facilitators before and after project training
Adoption	Characteristics of child health providers and parenting group facilitators (eg, age, sex, and education level) Number of child health providers and parenting group facilitators in each cluster Availability of space to implement intervention activities Support mechanisms for child health providers and parenting group facilitators Adoption of the intervention by clusters based on their contexts
Implementation	Fidelity: audit of intervention sessions, proportion of the content delivered as intended, and duration of each intervention activity Reasons for dropout and coping strategies among child health providers, parenting group facilitators, and caregivers Visit completion and contact hours among target caregivers Feasibility and acceptability
Maintenance	Proportions of child health providers and parenting group facilitators remaining engaged and dropping out Proportion of communities or townships remaining in the intervention Visit completion and dropout rates for target children and caregivers

a RE-AIM: Reach, Effectiveness, Adoption, Implementation, and Maintenance.

We will also conduct an explanatory qualitative study to identify the facilitators and barriers to project implementation by distinguishing differences between high-performing and low-performing clusters, which will be guided by the Capability, Opportunity, Motivation, and Behavior (COM-B) system [45]. The COM-B system posits that capability (eg, knowledge and skills), opportunity (eg, availability and power to fulfill a role), and motivation (eg, commitment to fulfilling a role) shape individual behavior. We aim to compare the differences in implementing behaviors and associated factors for both intervention service providers and service recipients between high-performing and low-performing clusters. To carry out this, we will identify 2 clusters in the intervention arm within each province (1 high-performing cluster and 1 low-performing cluster) based on differences in both the percentage of target children who attend parenting group activities and the percentage who receive clinical consultation services. To understand the natural development status in the control group, we will also select 1 control cluster with an average level of performance in each province. In each selected cluster, 7 stakeholders (1 township hospital director, 1 child health provider, 2 parenting group facilitators, and 3 caregivers) will be interviewed via semistructured in-depth interviews to explore differences in individual role characteristics between high-performing and low-performing clusters based on the COM-B system. In addition, we will observe the intervention services for each child health provider and parenting group facilitator on-site in the selected high-performing and low-performing clusters.

### Economic Study

We will compute the cost of the intervention and conduct an economic evaluation with an incremental cost-effectiveness analysis. For assessing the cost of the intervention, we will compute costs that are only related to the intervention. We will exclude costs related to providing usual primary health care services (control arm) and research costs related to intervention development and evaluation. We will follow the approach by Baek et al [46] and divide costs into start-up and recurrent costs. Start-up costs include electronic system development costs, material costs (eg, electronic devices) related to health checkups, costs of establishing ECD centers in the intervention arm (including equipment and toys), costs of recruitment (parenting group facilitators), and costs of training workshops. Recurrent costs can be divided into five categories as follows: (1) costs of health checkups in the intervention arm estimated with child health providers’ time input and unit salary; (2) costs of parenting group activities measured with parenting group facilitators’ time input and unit salary (as well as some nonnegligible session-specific material costs of parenting group activities); (3) costs of supervision at various levels in a tiered supervision system; (4) potential costs of material refill (eg, toys) and replacement of personnel (eg, due to attrition of parenting group facilitators); and (5) time opportunity costs for both service providers and households [46,47]. Taking advantage of our electronic survey system, we will be able to retrieve 2 key cost components (time inputs in categories 1 and 2) from the stored data, the collection of which would have been a significant challenge in a different setting. Unit analysis will calculate both the total intervention cost per child and the recurrent cost per child, and if needed, we may perform unit analysis controlling for the frequency of attending parenting group services. The incremental cost-eﬀectiveness ratios with and without nonrecurrent costs will be estimated by dividing either the total cost per child or the recurrent cost per child by the mean diﬀerence in eﬀects, based on our primary and secondary outcomes, where possible [48,49].

### Data Collection

For each child enrolled in this study, data will be collected from medical records, baseline and endline surveys, and monitoring during the intervention process, and all information will be integrated into a unified dataset via our electronic survey system (Table 5 and Multimedia Appendix 1).

**Table 5.** Data sources and collection approaches in the baseline and endline surveys.

Outcome of interest	Measurement tool	Data collection approach	Baseline	Endline
Primary outcome				
Children’s overall development	GSED^a^ combined short and long forms	Combined	✓	✓
Secondary outcomes				
Children’s overall development-long form	GSED long form	Onsite evaluation	✓	✓
Children’s overall development-short form	GSED short form	Interview	✓	✓
Children’s developmental delay	ASQ-3^b^ and PRIDE^c^	Self-report	✓	✓
Children’s anthropometric measurements, including length and weight (stunting rate and wasting rate)	Standardized tools (BEIGAO 0‐3 Years Lying Medical Checkup Gauge FSG-25-YE)	Onsite evaluation	✓	✓
Children’s hemoglobin levels in g/dL (anemia rate)	Hemoglobin tester (HemoCue HB 301)	Onsite evaluation	✓	✓
Feeding practices (correct diversity rate and correct frequency rate of complementary feeding and breastfeeding)	Age-adapted questions	Interview	✓	✓
Parenting capability, motivation, opportunity, and interactions with children among primary caregivers	Self-administered questionnaire	Interview	✓	✓
Parenting behavior of primary caregivers (integrated family parenting environment)	FCI^d^ scale	Interview	✓	✓
Mental health of primary caregivers	CED-S^e^	Self-report	✓	✓
Family discipline behaviors	MICS^f^	Self-report	✓	✓
Family function	APGAR^g^ assessment scale	Self-report	✓	✓
Children’s screen exposure	Self-administered questionnaires	Interview	✓	✓
Quality of life of primary caregivers	EQ-5D-5L questionnaire	Self-report	✓	✓
Parent-child interactions between the primary caregiver and child^h^	—^i^	Video	✓	✓
Respiratory infections in children^j^	Self-administered questionnaires	Self-report	—	—
Moderators				
Demographic information	—	Medical record	✓	—
Socioeconomic status	—	Interview	✓	✓
Participation in parenting services or early education programs	—	Interview	—	✓
Children’s major family changes during the intervention year	—	Interview	—	✓

a GSED: Global Scale of Early Development.

b ASQ-3: Ages and Stages Questionnaire third version.

c PRIDE: Parent-Reported Indicator of Developmental Evaluation for Chinese Children.

d FCI: Family Care Indicators.

e CED-S: Center for Epidemiology Depression Scale.

f MICS: Multiple Indicator Cluster Surveys.

g APGAR: Adaptation, Partnership, Growth, Affection, Resolve.

h The results of parent-child interactions will not appear in the main trial report article. The results will be published in a separate paper.

i Not applicable.

j The questionnaire survey on respiratory infections in children was conducted in April 2025, outside both the baseline and endline phases.

Children’s sex, birth date, family address, gestational age, and birth weight will be obtained from local medical records. During recruitment, child health providers in each community or township will identify the primary caregiver of each recruited child. The primary caregiver is defined as the person who is most familiar with the child and spends the most time with the child. This information will be recorded in the survey system by data management staff.

Child health providers will inform primary caregivers by telephone or through in-person visits to bring their children for the baseline survey. The baseline survey will be arranged in the community/township or county MCH hospital. The survey will begin with interviews to collect caregiver-reported measures and the GSED short form, progress to the GSED long form, and end with a physical examination. All interviewers will undergo a 1-day training program prior to the survey. For physical examination, the BEIGAO 0‐3 Years Lying Medical Checkup Gauge FSG-25-YE will be used for length and weight measurements. The HemoCue HB 301 will be used for hemoglobin testing. To ensure data quality for the GSED evaluation, a 5% sample will be parallel-scored by 4 GSED trainers to confirm >90% consistency on item scoring between trainers and assessors. In each county, the baseline survey will last for 9‐18 days, depending on the number of townships included. All families surveyed at baseline will be recontacted to undergo an endline survey 12 months later. With the exception of an additional satisfaction questionnaire, the duration, procedures, and measures will be identical to those at baseline. We will make up to 3 attempts to contact each family for follow-up before excluding them from the assessment.

Adherence data will be automatically recorded when families attend consultations during health checkups or participate in parenting group activities. In addition to utilization data, reasons for nonparticipation in ECD services and instances of loss of follow-up will be recorded by child health providers during routine supervision. During the endline evaluation, we will collect detailed information from families in both groups regarding participation in parenting services or early education programs throughout the study period.

### Statistical Analysis

The primary analysis will follow the intention-to-treat principle, which will include all recruited children in the randomized counties according to their original treatment allocation, irrespective of their subsequent adherence or how they actually received the intervention. We will present descriptive statistics for the demographic and socioeconomic status, the child nutrition status, child development, and parental measures at the individual level, as well as the number of pediatric health providers at the cluster level, along with appropriate summary statistics and their associated 95% CIs for all outcomes.

For child development, using the R package *dscore* [36], item responses from the GSED evaluation will be used to calculate each child’s D-score for the long and short forms, and this score serves as a unit of measurement on an interval scale representing child development, with a higher score indicating better overall development. The D-score will be further transformed into the DAZ with a normal distribution of scores (mean of 0 and variance of 1), which allows comparison between samples of different ages. For the primary outcome, we will use the combined-form DAZ, which is derived from all items included in both the long and short forms. For secondary analyses, the DAZ will be calculated separately for the long and short forms. For child growth, stunting and wasting rates will be calculated according to World Health Organization standards [50], using children’s anthropometric measures and age.

The generalized estimating equation (GEE) will be used to assess the population average intervention effect. The GEE approach models mean responses across all clusters. The GEE model will estimate the intervention effect for the child’s DAZ, adjusting for corresponding baseline outcomes, individual variables (child gender and maternal education), cluster variables (number of pediatric health providers and county), and variables that are significantly different at baseline where appropriate.

To understand whether the intervention is more effective for some families than others, predefined subgroup analyses for the primary outcome (DAZ) will be performed based on age group, maternal education level, and study counties. We will also conduct a mediation analysis using structural equation modeling to explore the role of parental behavioral change (group activity attendance rate) in the observed effects on child outcomes. Regarding missing data and loss to follow-up, we will apply the last observation carried forward approach, whereby the baseline value is carried forward to impute missing endline outcomes. To assess the robustness of the findings, sensitivity analyses will be conducted using alternative methods for handling missing data, including multiple imputation.

We will consider statistical significance at the 5% level and base our inferences on 2-sided *P *values and the associated 95% CIs for treatment effect estimates. All outcomes will be analyzed following study completion, and no interim analyses are planned for this study (Multimedia Appendix 3).

### Analysis of Process Evaluation

Both quantitative and qualitative data will be analyzed. Quantitative data will be summarized according to the RE-AIM framework and analyzed using appropriate statistical methods, including multiple linear regression for continuous outcomes, multiple logistic regression for binary outcomes, and multilevel modeling where nested structures exist. Qualitative data from semistructured interviews and focus group discussions will be analyzed using a thematic analysis approach. Transcripts will be imported into NVivo 14 (Lumivero) for data management. Two independent coders will initially generate codes from the data using an inductive-deductive approach, guided by the COM-B framework while also identifying emergent themes. Codes will be iteratively reviewed and grouped into candidate themes, which will be refined through team discussions to ensure consistent interpretation. Themes will then be categorized based on their roles as facilitators or barriers to implementation performance, particularly distinguishing between high-performing and low-performing clusters [51]. Illustrative quotations will be selected to support each theme (Multimedia Appendix 4).

To enhance reflexivity, the research team will document and discuss their perspectives and assumptions throughout coding and theme refinement to minimize bias in data interpretation. To ensure trustworthiness, strategies addressing credibility, dependability, confirmability, and transferability will be implemented, including double-coding of transcripts, peer debriefing, maintaining an audit trail of coding decisions, and providing thick contextual descriptions of participants and study settings.

### Ethical Considerations

The trial protocol has obtained ethical approval from the Institutional Review Board of Shanghai Children’s Medical Center Affiliated to Shanghai Jiao Tong University, School of Medicine (reference: SCMCIRN-K2024064-1 to SCMCIRN-K2024064-5). Written informed consent will be obtained from each participant’s caregiver. During data storage and analysis, all personally identifiable information will be removed in advance, and data will be anonymized, with child ID numbers serving as unique identifiers.

### Trial Management

We will establish a data management committee made up of independent members from our trial to ensure the highest safety and confidentiality of all participants. This committee will strictly follow established ethical guidelines, guaranteeing that all collected data are adequately stored and exclusively used for research purposes. Additionally, a trial steering committee, also led by independent trial members, will be created to oversee the direction and progress of the trial. To promote effective communication and collaboration, we will hold regular online meetings for both the data management committee and the trial steering committee. Furthermore, Professor FJ from the Shanghai Jiao Tong University School of Medicine will serve as the guarantor for this trial, and she will have complete access to the trial dataset. Finally, a trial management group will be established in Shanghai, consisting of 3 research associates from the Shanghai Jiao Tong University School of Medicine. This group will be responsible for managing the routine operational aspects of the trial, ensuring its smooth and successful implementation.

## Results

**Figure 2.**
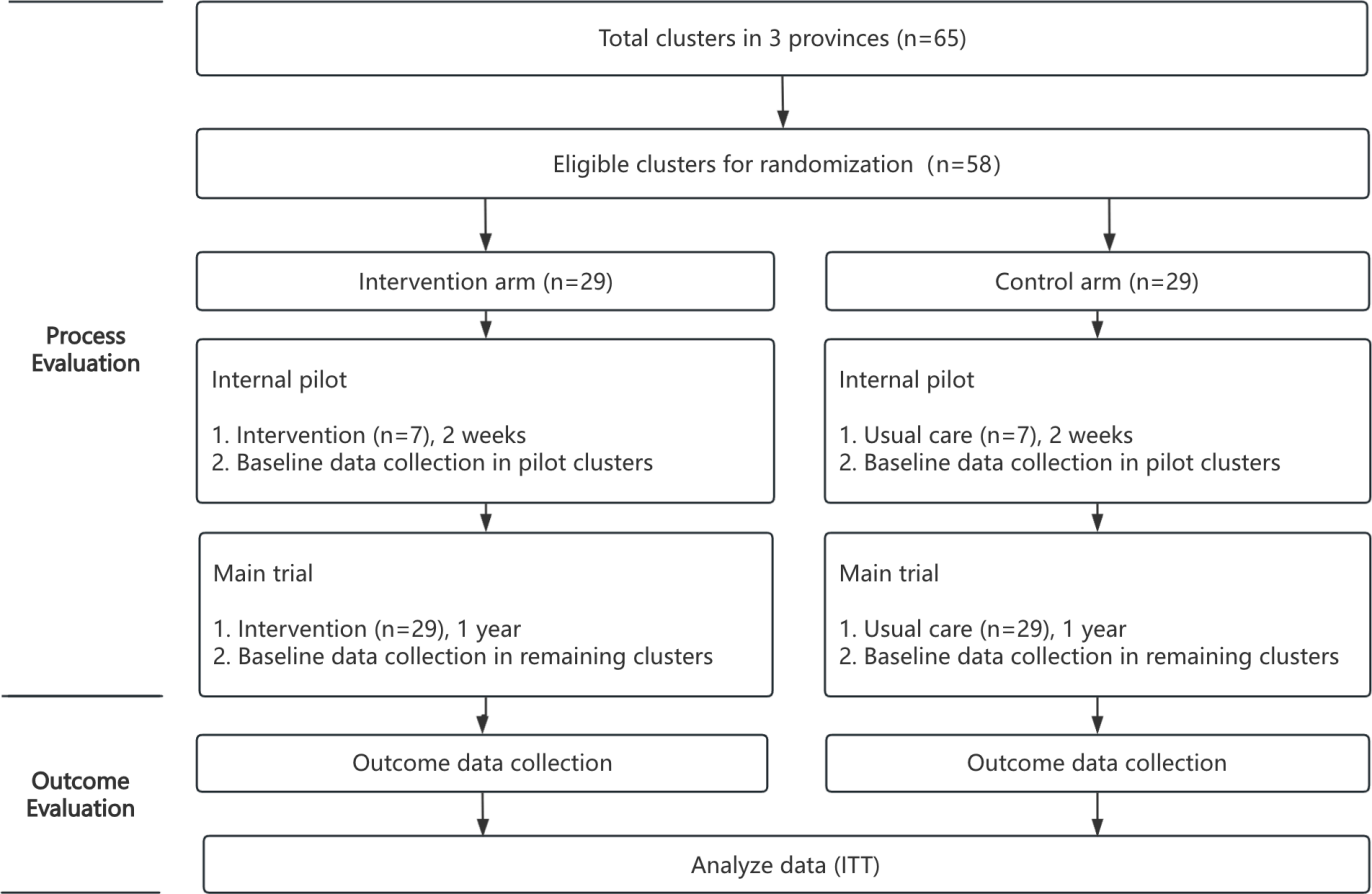
Study flow diagram. ITT: intention-to-treat analysis.

This study was funded in 2023 and registered in the Chinese Clinical Trial Registry (ChiCTR2400083465) on April 25, 2024. Preparation was from August 2023 to June 2024. Participant recruitment began on June 30, 2024, and concluded on September 14, 2024. A total of 1044 participants have been recruited for the trial. Data collection was completed on January 31, 2026. The study flow diagram is presented in Figure 2. The trial results will be reported through research articles and policy reports, and the findings of this study will be disseminated through peer-reviewed research articles, policy reports, and presentations at academic conferences.

## Discussion

### Anticipated Findings

This trial will generate critical evidence on the feasibility, effectiveness, and cost-effectiveness of integrating enhanced ECD services into China’s primary health care system. All evidence will inform China’s national policy to scale up the enhanced ECD program. The Government of China started an enhanced ECD program in 2013 in 4 pilot counties across 2 provinces, aiming to enhance nurturing care in disadvantaged rural areas [52]. The program expanded to 14 counties in 5 provinces in 2017 and to 29 counties across 10 provinces in 2019. During this process, techniques, including consultations, parenting group activities, and home visits, have been developed and adapted with each pilot. However, these components have only been evaluated individually in small-scale studies [48,49,53]. The effectiveness of implementing a comprehensive ECD service package through the MCH system across diverse socioeconomic regions has not yet been rigorously assessed. More importantly, a fully established implementation strategy remains lacking. For example, the training curriculum was not unified, and services were provided in various settings, including primary care facilities, village clinics, and kindergartens, by less organized personnel. Supervision and monitoring were lacking at the primary care level. Our current intervention builds upon these previous efforts by integrating them into a scalable, system-based approach and rigorously evaluating their combined impact. This study has designed an enhanced ECD service provision model that can seamlessly integrate with existing primary health care systems. As shown in Figure 1, the county-level MCH institute could provide training and supervision to primary care facilities, while the enhanced ECD services delivered in primary care could be reported and monitored by a superior MCH institute. Under China’s existing MCH system, governance—including health policy, financing, and regulation—can be implemented from the state level down to primary care.

### Strengths and Limitations

By using the GSED score as the primary outcome, this will be the first study to examine the effect of an enhanced ECD intervention on improving ECD at the population level. We will use an implementation science framework to inform the process evaluation to identify barriers and facilitators. The cost of enhanced ECD will be estimated to inform the financing strategy for implementing enhanced ECD across the entire population in China. We will use a multicenter approach to implement and evaluate this project across eastern, central, and western China, aiming to develop a strategy that can be applied in both developed and underdeveloped regions. This design also allows us to explore whether the intervention can help narrow the gap in ECD outcomes across regions with different socioeconomic statuses. Furthermore, this project will contribute to existing practices by exploring pathways for scaling up enhanced ECD initiatives in other developing countries.

One potential risk in this study is that parents may not attend the parenting group activities as scheduled. To mitigate this risk, we will establish a tiered supervision system to monitor the attendance rate for each child in the intervention arm. Child health providers will check the attendance rate weekly and remind families who have missed 2 consecutive parenting group activities. Additionally, county-level supervisors will monitor attendance rates for each township. For townships with persistently low attendance rates, an expert team will conduct on-site supervision at the ECD center and provide guidance to improve services. In cases where caregivers encounter difficulties in bringing children to the center, home visit parenting group activities will be offered as a supplement to regular parenting group sessions.

Several other limitations and challenges need to be noted. First, due to the nature of the intervention, child health providers and families will not be blinded to group assignment. This may introduce biases, such as differential behavior or care provision by providers, or changes in family engagement (eg, seeking out intervention activities despite being in the control group). These biases may be further compounded by the Hawthorne effect. However, this limitation is common in all ECD interventions at present. We aim to minimize this risk by ensuring consistent standards in training and supervision for regular health checkups, ensuring that primary health care provided by child health providers in both the intervention and control arms remains consistent. Second, by using a cluster-level intervention design, we have attempted to minimize the risk of contamination on the health provider side. Only child health providers in the intervention arm will receive training in ECD skills. However, as some communities or townships are adjacent to each other, there is a possibility that families in the control arm may be attracted to group activity sessions in the intervention cluster. To prevent contamination, we will implement an algorithm in the scheduling system for parenting sessions. If families in the control arm attempt to attend a session in the intervention cluster, the health workers will inform them that the session is fully booked. For ethical reasons, we will use a waitlist design for this trial. After evaluating the effects of the intervention, if effective, all clusters in the control arm will receive support to implement the enhanced ECD services within their primary health care systems, including infrastructure, equipment, toys, information systems, standardized training, and supervision. If the trial results show no significant difference, we will investigate potential implementation issues and discuss appropriate additional services for control areas. Third, we have excluded children who stay far from ECD centers, are younger than 6 months, and have a primary caregiver older than 65 years, as these have been identified as barriers to attending parenting group activities, limiting the generalizability of the results to those children. This may result in a sample that is more accessible and engaged than the broader target population, potentially overestimating feasibility and uptake. Our intervention is designed as a primary care-based model prioritizing population-level coverage and cost-efficiency, rather than replacing intensive approaches for high-risk groups. For remote and vulnerable families, more targeted strategies (eg, home visits and village-level service delivery) may be more appropriate. In this study, we will provide complementary home visits for families who cannot visit the centers for various reasons. In the future, with more skilled parenting group facilitators, this task could be shifted toward providing ECD services to these hard-to-reach families.

As China continues to advance and expand its ECD services, this project aims to inform national policy on ECD by offering standardized solutions in training, organization, personnel allocation, and stewardship within the primary health care system. The findings will not only guide health system policy but also provide critical evidence for financial investments across all levels of the government to support the scaling up of enhanced ECD services. Furthermore, it will inform policies to address regional disparities in child development outcomes and guide global efforts to scale ECD interventions.

## Supplementary material

10.2196/89106 Multimedia Appendix 1 Age-adapted consulting checklist.

10.2196/89106 Multimedia Appendix 2 Supervisor form.

10.2196/89106 Multimedia Appendix 3 Statistical analysis plan.

10.2196/89106 Multimedia Appendix 4 Additional details (program development, pilot testing, development of the eHealth platform for the intervention group, and summary of the interview/focus group guide).

10.2196/89106 Checklist 1 SPIRIT checklist.

## Abbreviations

ASQ-3: Ages and Stages Questionnaire third version
COM-B: Capability, Opportunity, Motivation, and Behavior
D-score: developmental score
DAZ: development-for-age z score
ECD: early childhood development
GEE: generalized estimating equation
GSED: Global Scale of Early Development
ICC: intracluster correlation coefficient
MCH: maternal and child health
PRIDE: Parent-Reported Indicator of Developmental Evaluation for Chinese Children
RE-AIM: Reach, Effectiveness, Adoption, Implementation, and Maintenance
SPIRIT: Standard Protocol Items: Recommendations for Interventional Trials
