# Supplementary material for: Evaluating the Effectiveness of an Enhanced Early Childhood Development Program Integrated Into Primary Health Care in China: Protocol for a Cluster Randomized Controlled Trial
Source: JMIR Res Protoc. 2026 May 27;15:e89106. doi: 10.2196/89106 (PMC13215665; doi:10.2196/89106)
Supplement: Multimedia Appendix 1 [file resprot-v15-e89106-s001.docx]

Appendix 1. Age-adapted consulting checklist

| **month** | **nutrition questions** | **stimulation questions** |
| --- | --- | --- |
| **6-month** | 1. Have you breastfed your child in the past 24 hours?  2. Have you given your child complementary food in the past 24 hours? Note: Complementary foods" refer to solid or semi-solid meals. This count excludes liquid milk (formula, cow's milk, goat's milk, yogurt, etc.).  3. Have you fed your child iron-rich foods (such as iron-rich animal-based foods/iron-fortified rice cereal) in the past 24 hours?  4. Do you supplement your child with vitamin D daily (including nutrients containing vitamin D)? | 1. Do you rarely play with your child?  2. Do you rarely talk to your child or tell them stories?  3. Are there fewer than 3 toys in your home (including homemade toys)?  4. Is there no parental involvement in caregiving, or are caregivers frequently changed? |
| **8-month** | 1.In the past 24 hours, have you fed your child breast milk? 2.How many times did you give your child complementary foods in the past 24 hours? Note: Complementary foods" refer to solid or semi-solid meals. This count excludes liquid milk (formula, cow's milk, goat's milk, yogurt, etc.). 3.In the past 24 hours, did you give your child staple foods (such as rice, noodles, grains, or tubers)? 4.In the past 24 hours, did you give your child meat, liver, blood products, fish, or shrimp? 5.In the past 24 hours, did you give your child eggs? 6.In the past 24 hours, did you give your child vegetables or fruits? 7.In the past 24 hours, did you give your child animal milk (e.g., cow's milk, goat's milk) or dairy products (e.g., yogurt, cheese)? 8.In the past 24 hours, did you give your child iron-rich foods (such as iron-rich animal-source foods or iron-fortified infant cereal)? 9.Do you give your child a vitamin D supplement (including supplements containing vitamin D) daily? | 1. Do you rarely play with your child?  2. Do you rarely talk to your child or tell them stories?  3. Are there fewer than 3 toys in your home (including homemade toys)?  4. Is there no parental involvement in caregiving, or are caregivers frequently changed? |
| **12-month** | 1.In the past 24 hours, did you breastfeed your child? 2.How many times did you offer complementary foods to your child in the past 24 hours? Note: Complementary foods" refer to solid or semi-solid meals. This count excludes liquid milk (e.g., formula, cow's milk, goat's milk, yogurt, etc.). 3.In the past 24 hours, did you give your child staple foods (e.g., rice, noodles, grains, or tubers)? 4.In the past 24 hours, did you provide your child with meat, organ meats (liver), blood products, fish, or shellfish? 5.In the past 24 hours, did you feed your child eggs? 6.In the past 24 hours, did you offer your child vegetables or fruits? 7.In the past 24 hours, did you give your child legumes or nuts in powdered or pureed form? 8.In the past 24 hours, did you provide other types of liquid milk to your child? (Note: "Liquid milk" refers to dairy products other than breast milk, such as formula, cow's milk, or goat's milk. 9.In the past 24 hours, did you feed your child iron-rich foods (e.g., iron-rich animal-source foods or iron-fortified infant cereal)? 10.Do you provide a daily vitamin D supplement to your child (including multinutrient supplements containing vitamin D)? | 1. Do you rarely play with your child?  2. Do you rarely talk to your child or tell them stories?  3. Are there fewer than 3 toys in your home (including homemade toys)?  4. Is there no parental involvement in caregiving, or are caregivers frequently changed? |
| **18-month** | 1.In the past 24 hours, did you feed your child breast milk and/or other liquid milk? Note: "Liquid milk" refers to non-breastmilk dairy (e.g., formula, cow's milk, goat's milk). 2.How many times did you offer complementary foods to your child in the past 24 hours? (Note: "Complementary foods" refers to solid/semi-solid meals. Excludes all liquid milk.) 3.In the past 24 hours, did you provide staple foods (e.g., rice, noodles, grains, tubers) to your child? 4.In the past 24 hours, did you feed your child meat, organ meats (liver), blood products, fish, or shellfish? 5.In the past 24 hours, did you give your child eggs? 6.In the past 24 hours, did you offer vegetables or fruits to your child? 7.In the past 24 hours, did you provide legumes or nuts in powdered/pureed form to your child? 8.In the past 24 hours, did you feed your child iron-rich animal-source foods (e.g., red meat, blood products, liver)? 9.Do you administer a daily vitamin D supplement (including multinutrient supplements) to your child? | 1.Do you rarely play games or engage in activities with your child? 2.Do you rarely talk to your child or tell them stories?  3.Are there no picture books at home? 4.Does your child’s daily screen time (including TV, phones, tablets, etc.) exceed 1 hour in total? |
| **24-month** | 1.In the past 24 hours, did you feed your child breast milk or other liquid milk? Note: "Liquid milk" refers to non-breastmilk dairy (e.g., formula, cow's milk, goat's milk). 2.How many times did you offer complementary foods to your child in the past 24 hours? Note: "Complementary foods" refers to solid/semi-solid meals. Excludes all liquid milk. 3.In the past 24 hours, did you provide staple foods (e.g., rice, noodles, grains, tubers)? 4.In the past 24 hours, did you feed your child meat, organ meats, blood products, fish, or shellfish? 5.In the past 24 hours, did you give your child eggs? 6.In the past 24 hours, did you offer vegetables or fruits? 7.In the past 24 hours, did you provide legumes or nuts in powdered/pureed form? 8.In the past 24 hours, did you feed your child iron-rich animal-source foods (e.g., red meat, blood products, liver)? 9.Do you administer a daily vitamin D supplement (including multinutrient formulations) to your child? | "1.Do you rarely play games or engage in activities with your child? 2.Do you rarely talk to your child or tell them stories?  3.Are there no picture books at home? 4.Does your child’s daily screen time (including TV, phones, tablets, etc.) exceed 1 hour in total?" |
| **30-month** |  | 1.Do you rarely play games or engage in activities with your child? 2.Do you rarely talk to your child or tell them stories?  3.Are there no picture books at home? 4.Does your child’s daily screen time (including TV, phones, tablets, etc.) exceed 1 hour in total? |
| **36-month** |  | 1.Do you rarely play games or engage in activities with your child? 2.Do you rarely talk to your child or tell them stories?  3.Are there no picture books at home? 4.Does your child’s daily screen time (including TV, phones, tablets, etc.) exceed 1 hour in total? |

All items are developed in accordance with the National Guidelines for Nutrition and Feeding Assessment Services for Infants and Toddlers (Trial Implementation) and the National Guidelines for Early Childhood Development Services (Trial Implementation) issued by China's National Health Commission (NHC).
