# Supplementary material for: Evaluating the Effectiveness of an Enhanced Early Childhood Development Program Integrated Into Primary Health Care in China: Protocol for a Cluster Randomized Controlled Trial
Source: JMIR Res Protoc. 2026 May 27;15:e89106. doi: 10.2196/89106 (PMC13215665; doi:10.2196/89106)
Supplement: Multimedia Appendix 2 [file resprot-v15-e89106-s002.docx]

Appendix 2. Supervisor form

| **1.Supervision Form Clinical Consultation** | | | | |
| --- | --- | --- | --- | --- |
| **Supervision Items** | | **Supervision Content** | **Supervision Method** | **Issues Identified** |
| **Organizational Management** | 1.Staffing | Increase the number of child healthcare personnel for the Early Childhood Development Project based on the growing population of children under 3 years old in the jurisdiction. | On-site inspection |  |
|  | 2.Staff Training | Participate in provincial/municipal training on project service standards and technical skills to ensure mastery of service protocols, technical competencies, and job requirements. | Written test; On-site interview. |  |
|  | 3.Clinic Setup | Dedicate a space of at least 10 square meters equipped with a desk, computer, examination bed, etc | On-site inspection |  |
| **clinical service** | 1.Nutrition and Feeding Assessment | Use the project system's "Nutrition and Feeding Assessment" questions to evaluate infants and young children monthly, assessing breastfeeding, complementary food introduction, dietary balance, and eating behaviors, with a focus on evaluating the types and frequency of complementary foods. | On-site observation of operations; Review of system records; Phone verification. |  |
|  | 2.Nutrition and Feeding Consultation and Guidance | Provide personalized nutrition and feeding consultation and guidance to caregivers based on the results of the nutrition assessment system. Use a printed calendar to provide anticipatory guidance on age-appropriate nutrition and feeding to all caregivers. | On-site observation of operations; Phone verification. |  |
|  | 3.Parenting Risk Screening | Initial Screening: Use the project system's "Parenting Risk Screening" questions to evaluate whether caregivers' parenting behaviors meet the elements of responsive caregiving, focusing on interactions with the child and identifying challenges in the caregiving process. Re-screening: If initial screening is positive, provide targeted guidance, implement a one-month family intervention, and conduct re-screening using the paper-based Parenting Risk Screening Form. Registration: Maintain an electronic Registry of Positive Parenting Risk Screening Results. | On-site observation of operations; Review of system records; Inspection of registration forms; Phone verification. |  |
|  | 4.Parenting Guidance | Provide personalized parenting guidance to caregivers based on the assessment system results. Use a printed calendar to offer anticipatory guidance on age-appropriate nutrition, communication, and play, with an emphasis on responsive caregiving skills. Guide caregivers to understand the Psychological and Behavioral Development Milestones for Children Under 3 Years Old to improve family parenting capacity. | On-site observation of operations; Phone verification. |  |
|  | 5. Home Visits | For children with two consecutive positive parenting risk screenings, conduct visits through home visits, phone calls, or video calls. The first follow-up must be an in-person home visit. Referral is required if parenting risks persist after three home visits. Record home visit information in the Home Visit Logbook (electronic version). | Review of records; Phone verification. |  |
| **Household Questionnaire Survey** | 1.Satisfaction | Caregiver satisfaction with child healthcare services | Random telephone interviews; On-site questionnaire surveys |  |
|  | 2.Family Child-Rearing Practices | Caregiver knowledge & skills in child-rearing (including nutrition/feeding, parenting risks, and psychosocial development awareness) | Random telephone interviews; On-site questionnaire surveys. |  |
| **Problem Rectification** |  | Carry out rectification and implementation of the issues identified in the last supervision, and achieve tangible results | On-site verification. |  |
| **Supervisor:** |  |  | **Supervised Unit** |  |
| **Organization Assessed** |  |  | **Supervised Personnel** |  |
| **Supervision Date** |  |  |  |  |

| **2.Supervision Form for Care Group Activities** | | | | |  |
| --- | --- | --- | --- | --- | --- |
| **Supervisor:** |  | **Supervision Date** |  | |  |
| **Supervision Items** | **Supervision Content** | **Supervision Method** | **Pass/Fail** | **Issues Identified** |  |
| **Station Management** | 1. The activity room is labeled "Early Childhood Development Activity Station." Information about the instructor, the class schedule, and system regulations are conspicuously displayed on the walls. | **On-site Check** |  |  |  |
|  | 2. Equipped with 1-2 (full-time/part-time) group facilitators who have completed infant and toddler care training organized by the municipal health department, obtained a training qualification certificate, and are certified to work. |  |  |  |  |
|  | 3. Activity regulations and job responsibilities are established and implemented. The environment is tidy, clean, and sanitary. |  |  |  |  |
|  | 4.Review documentation, including but not limited to: meeting minutes, early childhood development module reports, class schedules, activity record logs, and records of toy/teaching material usage and disinfection.。 |  |  |  |  |
|  | 5.For each activity, standardized use of the mini-program is required to complete processes such as sign-in/sign-out, and system-based questionnaires must be properly completed. |  |  |  |  |
|  | 6.Review the number of activity participations by infants/toddlers in the intervention group. Verify the early childhood development module reports. If participation did not occur, determine the reasons |  |  |  |  |
| **Activity Implementation** | 1.Time Management & Transitions Reasonable timing and natural transitions: Welcome (3-5 min), Health Education (5-10 min), Parenting Sharing (5-10 min), Parent-Child Interaction (20-30 min), Closing & Next Activity Announcement (3-5 min). | **Video Review / On-site Observation** |  |  |  |
|  | 2.Health Education Correct use of educational cards with accurate interpretation; content expanded appropriately, presented clearly (e.g., summarized via rhyming catchphrases). |  |  |  |  |
|  | 3.Parenting Sharing Review of previous activities; guided parent sharing; scientifically sound parenting Q&A and summaries; accurate content expansion. |  |  |  |  |
|  | 4.Parent-Child Activities Faithful execution of curriculum; thorough understanding of activities; correct demonstrations; guided practice for parents; logical activity summarization. |  |  |  |  |
|  | 5.Home Practice Guidance Emphasize importance of home practice to parents/caregivers; identify potential household challenges; provide actionable solutions. |  |  |  |  |
|  | 6.Communication Techniques Active listening (eye contact, nodding, smiling, affirmations, clarifying questions); timely responses (verbal/non-verbal); specific praise for positive parental behaviors. | **Field Observation** |  |  |  |
|  | 7. Responsive Caregiving Facilitation group facilitators guide parents in responsive interactions during activities; prioritize observation and coach parents on child engagement strategies. |  |  |  |  |
| **Parent Satisfaction** | 1.Parent Outcomes Knowledge acquisition, skill development, and satisfaction (environment/group facilitators). | **On-site Inquiry or Telephone Spot Check (2-3 parents)** |  |  |  |
|  | 2.Child Engagement Level of participation in activities. |  |  |  |  |
|  | 3.Parent Engagement Level of involvement during sessions. |  |  |  |  |
|  | 4.Feedback Presence of further suggestions. |  |  |  |  |
| **Strengths & Innovations** |  | | | |  |
|  |  |  |  |  |  |
|  |  |  |  |  |  |
|  |  |  |  |  |  |
|  |  |  |  |  |  |
| **Recommendations & Action Plan** |  | | | |  |
|  |  |  |  |  |  |
|  |  |  |  |  |  |
|  |  |  |  |  |  |
| **Organization Assessed** |  | | | |  |
| **Personnel Assessed** |  | | | |  |
