# Supplementary material for: Evaluating the Effectiveness of an Enhanced Early Childhood Development Program Integrated Into Primary Health Care in China: Protocol for a Cluster Randomized Controlled Trial
Source: JMIR Res Protoc. 2026 May 27;15:e89106. doi: 10.2196/89106 (PMC13215665; doi:10.2196/89106)
Supplement: Multimedia Appendix 3 [file resprot-v15-e89106-s003.docx]

# Appendix 3

# Statistical analysis plan

Contents

[1. Analysis Set 1](#_Toc226539386)

[2. Statistical methodology and statistical analyses 1](#_Toc226539387)

[2.1. General Considerations 1](#_Toc226539388)

[2.2. Demographic and Baseline Characteristics Variables 1](#_Toc226539389)

[2.3. Primary Outcome 1](#_Toc226539390)

[2.4 Secondary Outcomes 2](#_Toc226539391)

[2.5 Sensitivity Analyses 3](#_Toc226539392)

[2.6 Subgroup Analysis 3](#_Toc226539393)

[2.7 Additional Analysis 4](#_Toc226539394)

[3. Mock-up Tables 4](#_Toc226539395)

| Abbreviation | Full name |
| --- | --- |
| ECD | Early Childhood Development |
| GSED | Global Scale of Early Development |
| RE-AIM | Reach, Effectiveness, Adoption, Implementation and Maintenance framework |
| SF | Short Form |
| LF | Long Form |
| DAZ | Development-for-Age-z-score |
| CF | Combined Format |
| ASQ-3 | Ages and Stages Questionnaire third version |
| CED-S | Center for Epidemiology Depression Scale |
| MICS | Multiple Indicator Cluster Surveys |
| UNICEF | United Nations International Children's Emergency Fund |
| FCI | Family Care Indicators |
| CRCTs | Cluster Randomized Controlled Trials |
| SD | Standard Deviations |
| D-score | Child’s Developmental Score |
| PRIDE | Parent-Reported Indicator of Developmental Evaluation for Chinese Children |

# Analysis Set

Primary analysis will use an intention-to-treat approach, which will include all recruited children in the randomized counties according to their original treatment allocation, irrespective of their subsequent adherence or how they actually received intervention. For the per-protocol analysis, the “good adherence rule” was pre-defined and only include participants with good adherence to the intervention will be included.

# Statistical methodology and statistical analyses

## 2.1. General Considerations

The statistical analysis team will first clean the data and construct analysis datasets suitable for statistical analysis. The specific analysis plan is divided into 2 parts: comparison of baseline characteristics of participants and efficacy analysis. Continuous variables will be described as mean ± standard deviation for normally distributed variables and as median and range for non-normally distributed variables. Categorical variables are presented as frequencies and percentages. We will claim statistical significance at the 5% level and base our inferences on the two-sided p-values and associated 95% confidence intervals of the treatment effect estimates. All outcomes will be analyzed at the end of the study and no interim analyses are planned for this study. All analyses will be performed using SAS 9.4 (SAS Institute Inc., Cary, NC) and R (version 4.2 or higher).

## 2.2. Demographic and Baseline Characteristics Variables

We will present descriptive statistics on demographic and socio-economic status, child nutritional status, child development and parental measures at the individual level, and the number of health providers in pediatric section at the cluster level. Unless explicitly stated otherwise, descriptive statistics for normally distributed continuous variables are: n, mean, standard deviation, minimum and maximum. The assumption of distribution is checked. For non-normally distributed variables, median, IQR and range are used. Descriptive statistics for categorical variables are: n, percentage and total (N). Baseline homogeneity is presented as the absolute standardized difference.

## 2.3. Primary Outcome

For primary outcome, using the R package *dscore* [10], item responses from GSED will be calculated into each child’s developmental score (D-score) as a combined form, which is a unit of measurement with an interval scale representing the child development. A higher score represents better overall development. D-score will be further transformed into the Development-for-Age-z-score (DAZ) with a distribution of scores normally distributed with a mean of 0 and a variance of 1, which allows comparison between samples different ages.

The generalized estimating equations(GEE) will be adopted to assess the population average intervention effect. GEE models the mean responses across all clusters. The GEE model will estimate the intervention effect for the child’s DAZ score adjusting for the corresponding baseline outcomes, individual variables such as child gender, maternal education, cluster variables such as the number of health providers in the pediatric section, county, and variables which are significantly different at baseline when appropriate. The model could be expressed as

$g\left( \mu_{i} \right)=X_{ij}\boldsymbol{\beta}$**,**

where $g\left( \mu_{i} \right)$ is the identity link function, $\mu_{i}$ is the mean of DAZ score for $i$ cluster,$X_{ij}$ is the covariates matrix for the $j$ subject in the cluster $i$, $\boldsymbol{\beta}$ is the coefficient vector, where $\beta_{1}$ is in particular the coefficient for the intervention.

${Var(Y_{ij}|X}_{ij})=v(\mu_{ij})\phi$ is the conditional variance of $Y_{ij}$ (observed DAZ score) given $X_{ij}$ ,where $v$ is a known variance function of $\mu_{ij}$ and $\phi$ is error variance need to be estimated. An exchangeable structure used to describe within-cluster correlation. Missing outcome data were handled using the last observation carried forward (LOCF) approach, whereby, for participants with missing endline data, the baseline value was carried forward to impute the missing outcome measurements.

## 2.4 Secondary Outcomes

1. Children’s overall development measured by DAZ calculated from short form and long form separately.

A GEE will be used for analysis, respectively. similar to the analysis method of primary outcome DAZ score.

1. Children's developmental delay (evaluated by ages and stages questionnaire third version, ASQ - 3) and Parent-Reported Indicator of Developmental Evaluation for Chinese Children (PRIDE)
2. .A GEE with a logit link function will be used to estimate the intervention effect for probability of the children's developmental delay adjusting for the corresponding baseline outcomes, individual variables such as child sex, child age, maternal education and the number of health providers in pediatric section, and treating the within-cluster correlation as a nuisance with an exchangeable correlation structure. covariates similar to the primary outcome analysis will be adjusted.
3. Anthropometric measurements of children (weight ;length) and hemoglobin level (g/dl)

For weight, length and hemoglobin levels, GEE models will be used for analysis, respectively, similar to the analysis method of primary outcome DAZ score.

1. Feeding practices (evaluated by a checklist with 4 - 9 binary indicators in specific age groups for breastfeeding, diet diversity, complementary feeding, and food supplements)

We will use GEE models with a logit function as the link function to analyze binary variable feeding practices, in a similar way to the analysis of children's developmental delay.

1. Other secondary outcomes such as primary caregiver's mental health, parenting knowledge, skills, behavior, parent - child interaction, family function, family parenting environment, children's screen exposure, and primary caregiver's quality of life, the respiratory infections in children and so on.

For continuous outcomes (such as parenting knowledge, skills, behavior, parent - child Interaction, family parenting environment, primary caregiver's quality of life), a GEE model will be used, similar to the analysis of primary outcome DAZ score. The outcome variable is used as the dependent variable, and the intervention, relevant covariates (such as child gender，child age, maternal education level, baseline evaluation.) are included for analysis. For binary outcomes such as children's screen exposure, mental health of primary caregivers and family discipline behaviors, a GEE model with a logit function as the link function will be used to analyze respectively, and relevant covariates and cluster random effects will also be considered.

## 2.5 Sensitivity Analyses

For missing primary outcome, multiple imputation will be used based on baseline information including age, primary caregiver, maternal education, family annual income, singleton birth, and township. The per-protocol analysis will be conducted with the population only included the participants with good adherence to the intervention.

**2.6 Subgroup Analysis**

Pre-defined subgroup analyses will be performed for our primary outcome DAZ for the following groups

1. Age ( 6 to 11 months age group, 12 to 17 months age group, and 18 to 23 months age group).
2. Maternal education level (primary school and below, junior high school, junior college, bachelor degree or above, do not know / refuse to answer).
3. Study counties (Yiwu, Xinmi, Zhijin).

Table S1. The estimated statistical power for each subgroup analysis

| Subgroup analysis | Cluster per arm | Cluster size | Power |
| --- | --- | --- | --- |
| By age group (one third of original cluster size in each group) | 29 | 6 | 0.57 |
| By maternal education (half of original cluster size in each group) | 29 | 9 | 0.68 |
| By county (Yiwu) | 7 | 18 | 0.31 |
| By county (Xinmi) | 8 | 18 | 0.34 |
| By county (Zhijin) | 14 | 18 | 0.54 |

Power was estimated based on the following parameters: effect size (d) = 0.26 SD, intra-cluster correlation (ICC) = 0.06, and α = 0.05.

**2.7 Additional Analysis**

We will also conduct a mediation analysis using structural equation modelling to explore the role of parental behavioral change in observed effects on child outcomes. A GEE model will estimate the effect of parental behavioral change (group activities attendance rate) on the child's DAZ score using DAZ score as the dependent variable, parental behavioral change (group activities attendance rate) as the main explanatory variable, and other covariates such as baseline DAZ score, child's sex, child's age, mother's education, and the number of health providers in pediatric section. Interactions between independent variables will be tested and included in the model if significant.

# 3. Mock-up Tables

Table S2. Cluster screened and randomized by counties

|  |  | Intervention | Control | Total |
| --- | --- | --- | --- | --- |
| Yiwu |  |  |  |  |
|  | Screened |  |  |  |
|  | Screened failure |  |  |  |
|  | Randomized |  |  |  |
| Xinmi |  |  |  |  |
|  | Screened |  |  |  |
|  | Screened failure |  |  |  |
|  | Randomized |  |  |  |
| Zhijin |  |  |  |  |
|  | Screened |  |  |  |
|  | Screened failure |  |  |  |
|  | Randomized |  |  |  |

Table S3. Subject enrollment and analysis set

|  | intervention | control | Total |
| --- | --- | --- | --- |
| Screened |  |  |  |
| Screened failure |  |  |  |
| Enrolled |  |  |  |
| Received intervention (ITT) |  |  |  |
| Drop-out or bad adherence |  |  |  |
| Per-protocol |  |  |  |

Table S4. Baseline characteristics of participants

|  | Intervention (n=) | Control (n=) |
| --- | --- | --- |
| **Individual Level** |  |  |
| **Demographic And Social Economic Status** |  |  |
| Age, mean(SD) |  |  |
| Sex, no.(%) |  |  |
| Primary caregiver, no.(%) |  |  |
| Maternal education, no.(%) |  |  |
| Family annual income, no.(%) |  |  |
| Singleton, no.(%) |  |  |
| Study counties, no.(%) |  |  |
| YIWU |  |  |
| XINMI |  |  |
| ZHIJIN |  |  |
| **Baseline Evaluation** |  |  |
| D-score (CF),mean(SD) |  |  |
| D-score (SF),mean(SD) |  |  |
| D-score (LF),mean(SD) |  |  |
| DAZ(CF),mean(SD) |  |  |
| DAZ(SF),mean(SD) |  |  |
| DAZ(LF),mean(SD) |  |  |
| Children’s developmental delay, no.(%) |  |  |
| Anthropometric measurements (weight),mean(SD) |  |  |
| Anthropometric measurements (length),mean(SD) |  |  |
| Stunting, no.(%) |  |  |
| Wasting , no.(%) |  |  |
| Hemoglobin level, mean(SD) |  |  |
| Anemia , no.(%) |  |  |
| Correct diversity of complementary feeding, no.(%) |  |  |
| Correct frequency rate of complementary feeding, no.(%) |  |  |
| Breastfeeding, no.(%) |  |  |
| Parenting capability of primary caregiver, mean(SD) |  |  |
| Parenting motivation of primary caregiver, mean(SD) |  |  |
| Parenting opportunity of primary caregiver, mean no.(%) |  |  |
| Integrated family parenting environment, mean(SD) |  |  |
| Family discipline behaviors, no.(%) |  |  |
| Children's screen exposure, no.(%) |  |  |
| Mental health of primary caregivers, no.(%) |  |  |
| Quality of life of primary caregivers, mean(SD) |  |  |
| Family function, no.(%) |  |  |
| **Cluster level** |  |  |
| Number of health providers in pediatrics’ section, mean(SD) |  |  |
| Average years of education for child health providers, mean(SD) |  |  |

Table S5. Primary and secondary outcomes

|  |  |  | Unadjusted | | Adjusted | |
| --- | --- | --- | --- | --- | --- | --- |
|  | Intervention (n=) | Control (n=) | Difference or OR (95% CI) | *p* value | Difference or OR (95% CI) | *p* value |
| **Primary outcome** |  |  |  |  |  |  |
| DAZ(CF),mean(SD) |  |  |  |  |  |  |
| **Secondary outcomes** |  |  |  |  |  |  |
| DAZ(SF),mean(SD) |  |  |  |  |  |  |
| DAZ(LF),mean(SD) |  |  |  |  |  |  |
| Children’s developmental delay, no.(%) |  |  |  |  |  |  |
| Anthropometric measurements (weight),mean(SD) |  |  |  |  |  |  |
| Anthropometric measurements (length),mean(SD) |  |  |  |  |  |  |
| Stunting, no.(%) |  |  |  |  |  |  |
| Wasting , no.(%) |  |  |  |  |  |  |
| Hemoglobin level, mean(SD) |  |  |  |  |  |  |
| Anemia , no.(%) |  |  |  |  |  |  |
| Correct diversity of complementary feeding, no.(%) |  |  |  |  |  |  |
| Correct frequency rate of complementary feeding, no.(%) |  |  |  |  |  |  |
| Breastfeeding, no.(%) |  |  |  |  |  |  |
| Parenting capability of primary caregivers, mean(SD) |  |  |  |  |  |  |
| Parenting motivation of primary caregivers, mean(SD) |  |  |  |  |  |  |
| Parenting opportunity of primary caregiver, no.(%) |  |  |  |  |  |  |
| Integrated family parenting environment, mean(SD) |  |  |  |  |  |  |
| Family discipline behaviors, no.(%) |  |  |  |  |  |  |
| Children's screen exposure, no.(%) |  |  |  |  |  |  |
| Mental health of primary caregivers, no.(%) |  |  |  |  |  |  |
| Quality of life of primary caregivers, mean(SD) |  |  |  |  |  |  |
| Family function, no.(%) |  |  |  |  |  |  |

* DAZ(CF) indicates Development-for-Age-z-score. DAZ(LF) indicates Development-for-Age-z-score with a long form. DAZ(SF) indicates Development-for-Age-z-score with a short form. with a combined form.

*individual variables such as child sex, child age, maternal education and the number of health providers in pediatric section.

Table S6. The primary and secondary outcomes in different subgroups

| Variables | Subgroups | n | Unadjusted | | Adjusted | |
| --- | --- | --- | --- | --- | --- | --- |
|  |  |  | Difference (95% CI) | Difference (95% CI) | Difference (95% CI) | *p* value for interaction |
| age |  |  |  |  |  |  |
|  | 6-11 month |  |  |  |  |  |
|  | 12-17 month |  |  |  |  |  |
|  | 18-23 month |  |  |  |  |  |
| maternal education level |  |  |  |  |  |  |
|  | Primary school and below |  |  |  |  |  |
|  | Junior high school |  |  |  |  |  |
|  | Junior college |  |  |  |  |  |
|  | Bachelor degree or above |  |  |  |  |  |
|  | Do not know / refuse to answer |  |  |  |  |  |
| study counties |  |  |  |  |  |  |
|  | Yiwu |  |  |  |  |  |
|  | Xinmi |  |  |  |  |  |
|  | Zhijin |  |  |  |  |  |

*individual variables such as child sex, child age, maternal education and the number of health providers in pediatric section.

Table S7. Sensitivity analysis of intervention effect on primary outcome

| Data sets | Outcome | Intervention (n=) | Control (n=) | Unadjusted | | Adjusted | |
| --- | --- | --- | --- | --- | --- | --- | --- |
|  |  |  |  | Difference (95% CI) | *p* value | Difference (95% CI) | *p* value |
| Per-Protocol analysis set | DAZ(CF) |  |  |  |  |  |  |
| Imputation method - Multiple imputation | DAZ(CF) |  |  |  |  |  |  |
