# Supplementary material for: Evaluating the Effectiveness of an Enhanced Early Childhood Development Program Integrated Into Primary Health Care in China: Protocol for a Cluster Randomized Controlled Trial
Source: JMIR Res Protoc. 2026 May 27;15:e89106. doi: 10.2196/89106 (PMC13215665; doi:10.2196/89106)
Supplement: Multimedia Appendix 4 [file resprot-v15-e89106-s004.docx]

**Appendix 4.** Additional details (program development, pilot testing, development of the eHealth platform for the intervention group, and summary of the interview/focus group guide)

**1. Program development**

The core intervention components were designed by an expert team commissioned by National Health Commission of China. These components include:

1. Clinical Consultation: Utilizing a standardized checklist to identify risks related to nutrition and stimulation, and providing guidance to parents based on the Nurturing Care Framework.
2. Parenting Group Activity: Conducting bi-weekly, age-specific parenting sessions that offer health education and demonstrations on parent-child interactions, with a focus on responsive care.
3. Home Visit: Enhancing parenting skills for families with low attendance at parenting group sessions. These visits are conducted collaboratively by parenting group facilitators and village health workers. They also provide health examinations and consultations to families with low attendance at regular health check-ups.

These service components are summarized in the "Services" column of Table 1. The outpatient clinic has standardized supporting questionnaires and guidance materials, and parenting group activities and home visits are equipped with supporting play and educational toys.

**2.Pilot testing**

we conducted a preliminary test in Yiwu City two weeks prior to the trial's official commencement. A brief description is included in the "Pilot Stage" section of the main text. In detail, this small-scale pilot aimed to: a) test the feasibility of the assessment procedures and estimate the time required to complete assessments for one cluster of children, and b) allow townships that had already completed baseline assessments to immediately begin using the eHealth system to deliver services. This helped verify the eHealth platform's feasibility and allowed us to identify and rectify initial issues.

**3. Development of the eHealth platform for the intervention group**

1. A clinic information platform for Clinical Consultations. This includes modules for nutrition/feeding assessment and guidance, parenting risk screening and guidance. The system uses adaptive questioning, dynamically adjusting items based on caregivers’ responses to enhance screening precision. It also automatically generates personalized guidance tailored to identified needs. A key feature is the generation of a QR code summarizing assessment results and individualized recommendations for each consultation, enabling parents to conveniently scan and retain the information for later reference.
2. A system for scheduling and managing attendance Caregivers check in and out via QR codes (e.g., through WeChat), allowing accurate tracking of actual session attendance. In addition, before each session, caregivers complete a brief set of structured questions on their interactions with the child since the previous session (e.g., frequency of activities), enabling monitoring of participation and compliance.

**4. Summary of the interview/focus group guide**

Table. Topic Guide for Semi-Structured Interviews and Focus Groups

| **Theme** | **Example Questions** |
| --- | --- |
| **Implementation Context & External Opportunities** | - What factors in your organization support or hinder the program?- Are there resources, policies, or environmental aspects that influence implementation? |
| **Stakeholder Roles & Experiences** | - How do you perceive your role in implementing the program?- What skills or capacities help or limit your participation? |
| **Motivation & Engagement** | - What motivates you to engage with the program?- Are there challenges affecting your motivation or commitment? |
| **Feasibility** | - Is the program practical to implement?- Are there logistical or operational challenges? |
| **Acceptability** | - How acceptable is the program to you and other participants?- Are there aspects participants find difficult or inappropriate? |
| **Sustainability** | - What factors would support the program’s continuation?- Are there foreseeable barriers to sustaining implementation? |
| **Additional Comments** | - Any additional suggestions or lessons learned?- Highlights of successes or challenges? |

*The full interview/focus group guide is available upon request
